# The effect of different public health interventions on longevity, morbidity, and years of healthy life

**DOI:** 10.1186/1471-2458-7-52

**Published:** 2007-04-05

**Authors:** Paula Diehr, Ann Derleth, Liming Cai, Anne B Newman

**Affiliations:** 1 Departments of Biostatistics, University of Washington, Seattle, WA, USA; 2 Department of Health Services, University of Washington, Seattle, WA, USA; 3 Veterans Administration Center for Excellence in Research on the Older Adult, Puget Sound Health Care System, Seattle, WA, USA; 4 Office of Analysis, Epidemiology, and Health Promotion, National Center for Health Statistics, Centers for Disease Control and Prevention, Hyattsville, Md. USA; 5 Department of Medicine, University of Pittsburgh School of Medicine, USA; 6 Department of Epidemiology, Graduate School of Public Health, Pittsburgh, PA, USA

## Abstract

**Background:**

Choosing cost-effective strategies for improving the health of the public is difficult because the relative effects of different types of interventions are not well understood. The benefits of one-shot interventions may be different from the benefits of interventions that permanently change the probability of getting sick, recovering, or dying. Here, we compare the benefits of such types of public health interventions.

**Methods:**

We used multi-state life table methods to estimate the impact of five types of interventions on mortality, morbidity (years of life in fair or poor health), and years of healthy life (years in excellent, very good, or good health).

**Results:**

A one-shot intervention that makes all the sick persons healthy at baseline would increase life expectancy by 3 months and increase years of healthy life by 6 months, in a cohort beginning at age 65. An equivalent amount of improvement can be obtained from an intervention that either decreases the probability of getting sick each year by 12%, increases the probability of a sick person recovering by 16%, decreases the probability that a sick person dies by 15%, or decreases the probability that a healthy person dies by 14%. Interventions aimed at keeping persons healthy increased longevity and years of healthy life, while decreasing morbidity and medical expenditures. Interventions focused on preventing mortality had a greater effect on longevity, but had higher future morbidity and medical expenditures. Results differed for older and younger cohorts and depended on the value to society of an additional year of sick life.

**Conclusion:**

Interventions that promote health and prevent disease performed well, but other types of intervention were sometimes better. The value to society of interventions that increase longevity but also increase morbidity needs further research. More comprehensive screening and treatment of new Medicare enrollees might improve their health and longevity without increasing future medical expenditures.

## Background

The primary emphasis in public health is on health promotion and disease prevention [1], but the situations where this is the most effective approach are not always clear. It is important to understand which strategies provide the most benefit to society so that limited resources can be used effectively. There are many conceptual frameworks for the social and behavioral determinants of health, such as that proposed by the Institute of Medicine [1] or Evans and Stoddart [2]. There are individual-level theories about health interventions such as the Health Belief Model [3] and the Transtheoretical Model [4]. There are also community or group-level theories that include ecological perspectives [5], community organization [6], and social marketing [7]. None of these theories, however, directly addresses the orientation toward prevention versus treatment for different populations [8]. That is, whether it is more effective to keep healthy persons healthy, to return sick persons to health, to keep sick persons from dying, or to keep healthy persons from dying.

**Figure 1:**
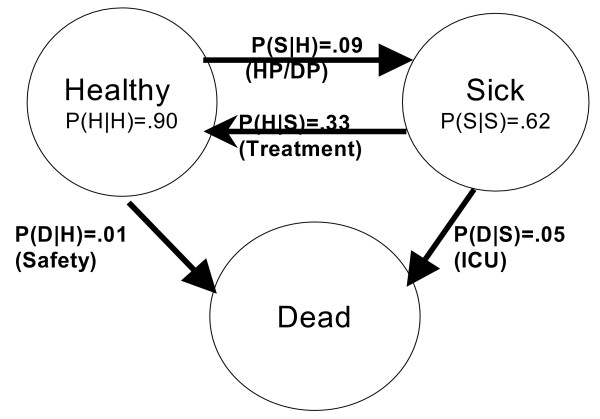
**One-Year Transitions Among Three Health States for Age 65**. P(A | B) is the probability of being in state A at age 66 for a person who is in state B at age 65. For example, P(S|H) is the probability that a healthy 65-year-old will be sick at age 66. The quantities in parentheses represent the generic interventions that would affect the relevant transition probability, as follows. HP/DP is health promotion and disease prevention, which affects the probability that healthy persons become sick. Treatment makes sick persons healthier. The ICU reduces the probability that sick persons die. The Safety intervention reduces the chance that healthy persons die.

Figure 1 represents "the public" as belonging to one of three states: healthy, sick, or (when followed over time) dead. The arrows indicate that persons can change states. The probabilities of transitioning to the various states one year later are shown for age 65. For example, using standard probability notation, P(S|H) = .09 indicates that the probability of a 65-year-old person being sick next year, given that he is healthy this year, is .09. In Figure 1, health promotion and disease prevention can be thought of primarily as decreasing the probability that healthy persons become sick, P(S|H). However, decreasing the probability that healthy or sick persons die (P(D|H) or P(D|S)), or increasing the probability that sick persons return to health (P(H|S)) would also improve the health of the public. How might these approaches differ in achieving public health goals?

Figure 1 may be thought of as a system with three states, healthy, sick and dead. The status of a system at any time is completely defined by its initial conditions (the number of healthy and sick persons at baseline) and the probabilities of transition to a different state. Public health interventions may thus attempt to improve health by changing the initial conditions, by modifying the transition probabilities, or both.

Initial conditions could be improved by moving some of the persons from sick to healthy at baseline (referred to later as the **One-Shot **intervention). An intervention could also aim to change the transition probabilities at each year of age. Such an intervention might decrease P(S|H), the probability that healthy persons become sick, through health promotion or disease prevention programs (**HP/DP**). Improved methods of and access to treatment can increase P(H|S), the probability that sick persons return to health (**Treatment**). Interventions such as improved ICU care may lower P(D|S), the probability that sick persons die (**ICU**). Finally, interventions to improve traffic, gun, or workplace safety could reduce P(D|H), the probability of death for healthy persons (**Safety**). One might also combine interventions, such as **HP/DP + One-Shot**, or **HP/DP + ICU**. Such interventions are likely to have different effects on longevity or years of life (**YOL**), years of healthy life (**YHL**), morbidity or years of sick life (**YSL**), and on medical expenditures.

The intervention types are defined formally in Table 1, which provides an algebraic, a text, and a mnemonic description for each type of intervention. The One-Shot intervention moves all of the sick persons to the healthy state, only once, at baseline. To allow for less potent interventions, we define One-Shot as an intervention that moves 100*λ% of the sick persons healthy at baseline only, where the parameter λ has a value less than or equal to one. The four interventions that improve the transition probabilities at every age are described algebraically as a function of a parameter, α, which is the amount of "improvement" in the relevant age-specific transition probability. For example, HP/DP is an intervention that multiplies P(S|H) by (1-α); to achieve a 10% improvement, α = 0.10, and P(S|H) is multiplied by 0.9, thus lowering the probability that a healthy person becomes sick by 10% at every age.

**Table 1:** Interventions, Parameters, and Terms

**Algebraic**	**Descriptive**	**Mnemonic Label**
**Interventions**		
No Intervention	No Change	Status Quo
Change the Initial Conditions:		
Let π' be π + λ(1 - π)	Move 100*λ% of the sick persons to healthy, at baseline only.	One-Shot
Improve the Probabilities:		
P(S|H) * (1-α)	At every age, lower the probability of a healthy person getting sick by a factor of (1-α), perhaps through such health promotion and disease prevention activities as smoking prevention programs or vaccinations	HP/DP
P(D|H) * (1-α)	At every age, lower the probability that a healthy person dies, perhaps by improving automobile, workplace, or gun safety.	Safety
P(D|S) * (1-α)	At every age, lower the probability that a sick person dies, perhaps by improving intensive care units.	ICU
P(H|S) * (1+α)	At every age, increase the probability that a sick person becomes healthy, possibly by improving treatment.	Treatment
		
**Parameters**		
λ	Proportion of sick persons moved instantaneously to the healthy state by the One-Shot intervention (usually set to 1.0)	
α	Amount of improvement to a transition probability (usually set to 0.1)	
β	Worth to society of an additional YSL divided by worth of an addition YHL (usually set to 0.5)	Relative Worth
π	Proportion who are healthy at baseline (usually set to 0.98 for birth cohort and 0.80 for retiree cohort)	
		
**Outcomes**		
YHL	Years of healthy life (years in excellent, very good, or good health)	YHL
YSL	Years of sick life (years in fair or poor health)	YSL
YHL + β*YSL	Worth-adjusted years of life	Worth

The names given to the intervention types do not refer to real interventions, but were chosen to help readers remember the intervention's primary feature. Consider a public health intervention that delivers antibiotics to a population. If the antibiotics are administered at one time only to a subset (possibly all) of the sick persons, with the goal of making 100*λ% of the sick persons immediately healthy, that would be a One-Shot intervention, with its strength measured by λ. If antibiotics are provided every year to a subset of the sick persons, with the goal of increasing the proportion of sick persons who are healthy one year later by a factor of (1+α), that would be a Treatment, with its strength measured by α. Antibiotics given every year to a sample of the sick persons to lower their probability of dying would be an ICU-type intervention. And antibiotics administered every year to a sample of the healthy persons would be a HP/DP or a Safety-type intervention depending on whether the primary goal was to prevent sickness or to prevent death. The antibiotic program would thus be classified differently depending on the targets, the timing and the major intended effect. More detailed examples are presented in the discussion section.

The goal of this paper is to compare the effects of the different types of intervention strategies on years of life, years of healthy life, and years of sick life (YOL, YHL, YSL) and on medical expenditures. We hypothesized that the HP/DP intervention would perform well, because prevention is the preferred strategy in public health. There may be situations, however, where prevention is not the best approach, because the effectiveness of an intervention depends on the nature and strength of the intervention, on the initial health and age of the target population, and on the value that society places on an additional year of sick life.

## Methods

### Health states and transition probabilities

We defined "healthy" as being in excellent, very good, or good health and "sick" as being in fair or poor health. Age-specific transition probabilities among the states were calculated from three large datasets, as explained in Appendix 1 and in more detail elsewhere [9]. Multi-state life tables were calculated from the transition probabilities. Based on initial conditions (the number of healthy and sick persons at baseline) and the transition probabilities, the life table provides estimates of the future years of healthy life (years spent in the healthy state) from baseline to age 100. An example of such calculations is given in Appendix 1. National estimates of the proportion of older adults who were healthy or sick at ages 0 and 65 came from the National Health Interview Survey [10]. Data on medical expenditures by age and health state were estimated from MEPS data collected in 2002, using the MEPSnet software [11]. These data were used to estimate future medical expenditures for each intervention.

### Interventions

We examined the performance of the types of intervention listed in Table 2. The "Status Quo" intervention made no change and is the basis for comparison. "One-Shot" is an intervention that moves 100*λ% of the sick persons to the healthy category at baseline only (λ is set to 1 in most of this paper). We also defined four hypothetical interventions, each of which affects exactly one of the transition probabilities in Figure 1. We calculated the effect of "improving" each of the transition probabilities by 100*α%. This improvement is defined as either multiplying P(H|S) by 1+ α (to increase the probability of recovery), or multiplying P(S|H), P(D|H), or P(D|S) by 1-α (to decrease the probability of getting sick or dying). In most of this paper, α is set to 0.10. We also evaluated the two combined interventions shown in Table 2, and compared each intervention to the "Status Quo" intervention.

**Table 2:** Outcomes* by Baseline State by Cohort

	**All Healthy at Baseline**			**All Sick at Baseline**			US Distribution at Baseline **		
**1**	**2**	**3**	**4**	**5**	**6**	**7**	**8**	**9**	**10**
**Intervention**	**YHL**	**YSL**	**YOL**	**YHL**	**YSL**	**YOL**	**YHL**	**YSL**	**YOL**
	**Birth Cohort**								
									
**Status Quo**	67.87	9.51	77.38	64.52	9.94	74.46	67.80	9.52	77.33
**One-Shot**	67.87	9.51	77.38	67.87	9.51	77.38	67.87	9.51	77.38
**HP/DP**	68.97	8.81	77.78	65.57	9.27	74.84	68.90	8.82	77.72
**Treatment**	68.72	8.96	77.69	65.55	9.31	74.86	68.66	8.97	77.63
**ICU**	68.20	9.77	77.97	65.09	10.23	75.32	68.14	9.78	77.92
**Safety**	68.24	9.63	77.87	64.87	10.06	74.92	68.17	9.64	77.81
**HP/DP+One-Shot**	68.97	8.81	77.78	68.97	8.81	77.78	68.97	8.81	77.78
**HP/DP+ICU**	69.29	9.05	78.34	66.13	9.54	75.67	69.22	9.06	78.28
									
	**Retiree Cohort**								
**Status Quo**	13.12	4.28	17.40	10.44	5.45	15.90	12.58	4.52	17.10
**One-Shot**	13.12	4.28	17.40	13.12	4.28	17.40	13.12	4.28	17.40
**HP/DP**	13.62	4.02	17.64	10.84	5.24	16.09	13.07	4.26	17.33
**Treatment**	13.43	4.12	17.55	10.94	5.20	16.14	12.93	4.34	17.27
**ICU**	13.30	4.50	17.79	10.71	5.71	16.43	12.78	4.74	17.52
**Safety**	13.36	4.38	17.74	10.64	5.53	16.17	12.82	4.61	17.43
**HP/DP+One-Shot**	13.62	4.02	17.64	13.62	4.02	17.64	13.62	4.02	17.64
**HP/DP+ICU**	13.80	4.22	18.01	11.12	5.49	16.60	13.26	4.47	17.73

* Years of healthy life, years of sick life, and Years of life due to a 10% improvement (α = .10) using the listed intervention. For One-Shot, the intervention makes everyone healthy at baseline (λ= 1.0 and α is irrelevant). For HP/DP+One-Shot, everyone is healthy at baseline and α = .10 for the HP/DP intervention).

** U.S. distribution at baseline is assumed to be 98% healthy at birth (π = .98) and 80% healthy at age 65 (π = .80). Column 8 is calculated as π*(column 2) plus (1 - π)*(column 5).

### The worth of an additional YHL or YSL

Consider two hypothetical interventions, A and B. Intervention A produces 4 additional years of healthy life and no additional years of sick life, while B produces 3 additional YHL and 2 additional YSL. Which intervention is better? The answer depends on the worth to society of an additional YHL or YSL. Worth might be measured in dollars (perhaps based on lost productivity or on higher medical expenditures for sick persons), or in some other way. Suppose we knew that an additional YHL was worth 100 "units" to society, and an additional YSL was worth 0; then intervention A would be preferred because it provided 400 units of worth compared to 300 for intervention B. Alternatively, if a YSL was worth 50, the two interventions would be equivalent because both would provide 400 units of worth. If a YSL was worth 100, there would be no distinction between YHL and YSL, and Intervention B would be preferred. We do not know the values for absolute worth (or even the units in which it should be measured), but we can think productively about the relative worth of a YSL and a YHL. Let β be the ratio of the worth of an additional YSL to the worth of a YHL. In the 3 cases above, β = 0/100 = 0, = 50/100 = 0.5, and = 100/100 = 1.0, respectively.

The best intervention will provide the most incremental worth to society for a given input. We assume that the worth of future years of life is K*(YHL + β*YSL) worth-adjusted years, where β is a number less than or equal to 1 and K is some constant that can be ignored with no loss of generality. If β = 1, future worth is YHL+1*YSL = YOL; society is indifferent to whether the person is in the healthy or sick state, and would seek to maximize life expectancy. If β = 0, future worth = YHL+0*YSL=YHL; society is indifferent to the sick and dead states, and the intervention that maximizes years of healthy life would provide the most worth. Negative values of β imply that sickness is a state worse than death [12]. We examined a range of β between -0.25 and 1.0. For each pair of interventions we calculated the intervention costs at which one intervention would be more cost-effective than the other, as explained below.

### Analysis

We first estimated the effect of each intervention, with λ = 1.0 and α = 0.10. For the HP/DP+One-Shot intervention, we moved all sick persons to the healthy state at baseline and also improved P(S|H) by α. For HP/DP+ICU, we improved both P(S|H) and P(D|S) by α. We estimated the YOL, YHL, and YSL for a cohort of size 100,000 at baseline, using multi-state life table software implemented in Stata [13]. This was done for both a Birth cohort (from age 0 to 100) and a Retiree cohort (from age 65 to 100). We also estimated average lifetime medical expenditures as the number of persons projected to be in each health state at each age multiplied by the average medical expenditure for that state and age, summed and divided by 100,000.

### Standardizing the comparisons

A comparison of interventions requires that we standardize the input or the output. For example, when interventions are compared on their cost per quality-adjusted life year, cost is the input and QALY is the output. Here, we will use incremental worth to society (improvement in worth-adjusted years of life) as the output, and account for input in two ways. We first examine the amount of output produced with a fixed input, α. Because One-Shot is not a function of α, we also examine the size of input (α) required to produce a fixed output, defined as the same output as the One-Shot intervention. This is explained in more detail below.

## Results

**Figure 2:**
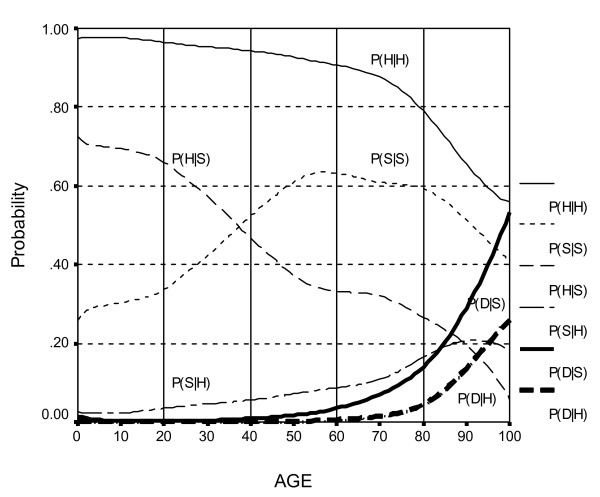
One-year Transition Probabilities.

Figure 2 shows the transition probabilities from age 0 to 100. These were estimated from three large longitudinal datasets, as explained in Appendix 1 and are listed in more detail elsewhere [9]. For example, the topmost line shows the probability that a person who is healthy at the age on the X axis will be healthy one year later, (P(H|H)). The lowest lines are the probabilities of getting sick or dying. The probabilities are quite favorable below about age 40, but after that the probability of remaining or becoming healthy declines and the probability of sickness or death increases. The interventions that improve the probabilities would raise the P(H|S) line by 10%, or lower the three bottom lines by 10%.

**Figure 3:**
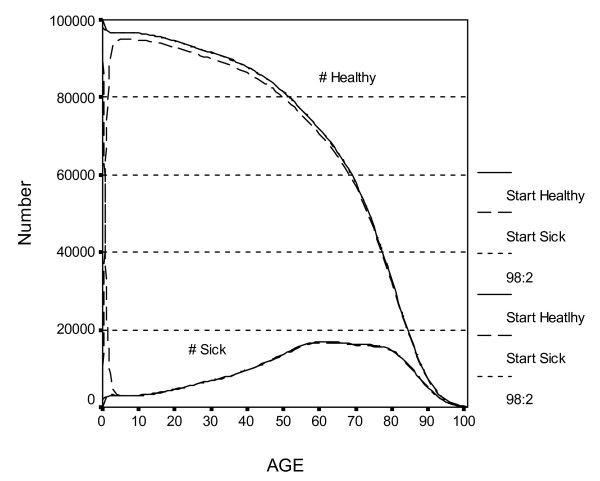
Estimated # of healthy and sick persons over time in Birth cohort (All healthy, All sick, or 98:2 ratio at Birth)*. *The topmost solid line is the number healthy when all were healthy at baseline, and the lower solid line is the number sick when all were healthy at baseline. The two dashed lines represent the number healthy and sick when all were sick at baseline. A third pair of dotted lines represent the number healthy and sick when 98% were healthy at baseline, but cannot be seen in this figure because they are essentially identical to the solid lines.

Figure 3 shows the number of persons in a Birth cohort who are predicted to be healthy or sick at each age. (The number dead is not shown). The solid lines represent a Birth cohort of 100,000 persons where everyone was healthy at birth; the upper line is the number who are healthy over time and the lower line solid line is the number who are sick. The dashed lines represent a cohort where everyone was sick at birth. The One-Shot intervention modifies the percent initially healthy or sick. Note that even in the "all sick at birth" cohort, most persons are healthy after a few years because P(H|S) is high at the younger ages (see Figure 2). Importantly, until about age 80 the number of sick persons is small relative to the number who are healthy. This will have consequences for the effectiveness of the various interventions.

The areas under the two "# healthy" curves are the expected years of healthy life (YHL), and differ for the two cohorts primarily because of the differences near age zero. The line labeled "Status Quo" in Table 2 (explained below) indicates that YHL is 67.87 years if everyone is healthy at birth, and 64.52 years if everyone is sick at birth. The area under the "# sick" curves is years of sick life (YSL) or morbidity. A third pair of (dotted) lines represents the number healthy and sick if 98% of the population is healthy at birth, similar to national estimates. In Figure 3, these lines are virtually indistinguishable from the "all healthy at birth" lines. YHL is 67.80 years.

**Figure 4:**
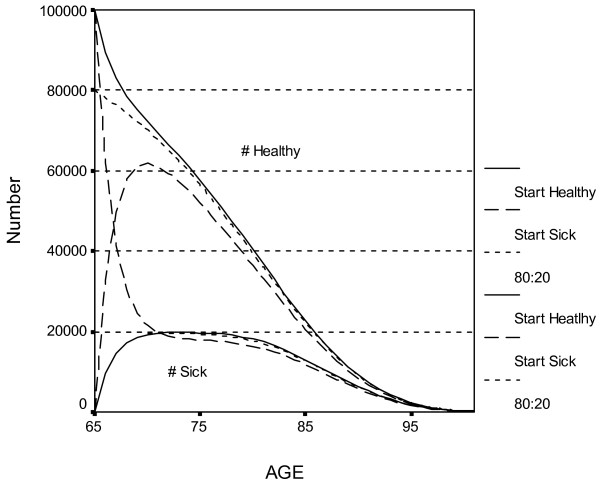
Estimated # of healthy and sick persons over time in Retiree cohort (All healthy, All sick, or 80:20 ratio at Age 65)*. *The topmost solid line is the estimate number of healthy persons when all were healthy at age 65, and the lower solid line is the number sick when all were healthy at age 65. The two dashed lines represent the number healthy and sick when all were sick at age 65. A third pair of dotted lines represent the number healthy and sick when 80% were healthy at baseline.

Figure 4 presents the same information, but for the Retiree cohort. As before, the two solid lines are the number healthy (upper line) and sick (lower line) when all are healthy at age 65. The two dashed lines are the number healthy and sick when all are sick at age 65. The two dotted lines reflect the number sick and healthy when 80% are healthy and 20% are sick at age 65, similar to national estimates. Note that the initial conditions (all sick versus all healthy versus 80% healthy at age 65) are more important than they were in Figure 3. The number of healthy people eventually becomes similar for all initial conditions, but it takes longer than in Figure 3. It is clear that the areas under the three top curves are different. YHL is estimated as 13.12 years if all are healthy at age 65, 10.44 years if all are sick, and 12.58 years if 80% are healthy.

Table 2 shows the estimated years of healthy life, years of sick life, and years of life for each intervention, by cohort, and depending on whether the cohort was all healthy (columns 2–4) or all sick (columns 5–7) at baseline. For example, the first line shows that under the Status Quo, persons in the Birth cohort who are healthy at birth average 67.87 healthy years and 9.51 sick years, summing to 77.38 years of life (life expectancy). Values for persons sick at baseline are shown next, and are less favorable (e.g., only 74.46 YOL). In columns 8–10, the percent healthy and sick at baseline are set to the national values: 98% initially healthy and 2% sick for the Birth cohort, and 80% healthy and 20% sick for the Retiree cohort. For example, for the Birth cohort, Column 8 is .98*(column 2) plus .02*(column 5).

The second line of Table 2 shows results for the One-Shot intervention. Note that the outcomes for the initially healthy cohort are identical to the Status Quo outcomes (because One-Shot did not affect those already in the healthy state) and that the outcomes for the sick cohort are identical to those for the initially healthy cohort, because all sick persons were moved to the healthy state at baseline. Similarly, results for HP/DP+ One-Shot are the same as HP/DP alone when all are healthy at baseline. Note that all of the interventions produce more YHL and YOL than the Status Quo, in both the Birth and Retiree cohorts.

### Incremental change in YHL, YSL, YOL, and medical expenditures

Columns 2, 3, and 4 of Table 3 are the same as the last three columns of Table 2, except that the value for the Status Quo has been subtracted from each line. For example, dYHL (difference in YHL) is 0 for the Status Quo intervention, by definition. The One-Shot intervention achieved 0.067 additional years of healthy life in the Birth cohort, which is the entry in the second row of Table 3. One-Shot also decreased YSL (years of sick life, or morbidity) by 0.009 years and increased YOL (survival) by 0.059 years. The effects of the One-Shot intervention were larger in the Retiree cohort. Column 5 shows the estimated difference in future medical expenditures attributable to the intervention, in dollars. All of the interventions improved YHL and YOL (dYHL and dYOL are always positive), although the improvement was not usually large. The Safety and ICU interventions increased both morbidity (dYSL) and medical expenditures (d$), while the other interventions decreased them. HP/DP + ICU decreased YSL but increased medical expenditures nonetheless. The entries in columns 2–5 of Table 3 were calculated for α = .10. However, we discovered that the amount of change in YHL, YSL, and YOL was approximately a linear function of α through the origin for |α| < .3 (except for One-Shot and HP/DP + One-Shot which do not depend specifically on α). This simplification is used later on to extend the standard configuration to other values of α.

**Table 3:** Improvements in Outcomes for the Interventions (10% improvement)

**1**	**2**	**3**	**4**	**5**	**6**	**7**
**Intervention**	**dYHL**	**dYSL**	**dYOL**	**d$**	**Worth (β = .5)**	**Required α**
	**Birth cohort**					
						
**Status Quo**	0	0	0	0	0	
**One-Shot**	0.067	-0.009	0.059	-122	0.0625	0
**HP/DP**	1.098	-0.700	0.398	-1283	0.7480	0.0084
**Treatment**	0.857	-0.555	0.303	-1070	0.5795	0.0108
**ICU**	0.334	0.258	0.592	4943	0.4630	0.0135
**Safety**	0.368	0.119	0.486	3404	0.4275	0.0146
**HP/DP + One-Shot**	1.166	-0.710	0.456	-1407	0.8110	0
**HP/DP + ICU**	1.420	-0.464	0.956	3352	1.1880	0.0053
						
	**Retiree cohort**					
						
**Status Quo**	0	0	0	0	0.0000	
**One-Shot**	0.535	-0.234	0.301	-2	0.4180	0
**HP/DP**	0.485	-0.252	0.233	-8	0.3590	0.1164
**Treatment**	0.346	-0.178	0.167	6	0.2570	0.1626
**ICU**	0.197	0.222	0.420	4079	0.3080	0.1357
**Safety**	0.237	0.092	0.329	2676	0.2830	0.1477
**HP/DP + One-Shot**	1.041	-0.497	0.544	-8	0.7925	0
**HP/DP + ICU**	0.677	-0.046	0.632	3846	0.6540	0.0639

Columns:

1: Name of intervention

2: dYHL=YHL for the row intervention minus YHL for the Status Quo

3: dYOL=YOL for the row intervention minus YOL for the Status Quo

4: dYSL=YSL for the row intervention minus YSL for the Status Quo

5: d$ = Total medical expenditures for the row intervention minus expenditures for the Status Quo.

6: Incremental worth-adjusted years of life (worth) of the row intervention minus worth of the Status Quo, calculated as dYHL + .5*dYSL.

7: Level of α required for the row intervention to provide as much incremental worth as the One-Shot intervention, calculated as the column 6 entry for One-Shot divided by the column 6 entry for the row intervention, multiplied by 0.10.

### Relative Effectiveness of an Intervention

Interventions differ in their effect on YOL, YHL, and YSL. We assume that the incremental worth to society of an intervention is K*(dYHL + β* dYSL), where K is a constant that can be ignored here, and β is the relative worth of an additional YSL. First, assume that β = 0.5 (that an additional YSL is worth half as much to society as an additional YHL). Column 6 of Table 3 shows the incremental number of worth-adjusted years for each intervention, calculated as dYHL+.5*dYSL. In the Birth cohort the two combined interventions provide the most worth, followed by HP/DP. Results are similar for the Retiree cohort except that One-Shot is the best simple intervention.

Worth to society may also be calculated for other values of β (see Appendix 2). For example, if β = 0, the worth is simply dYHL, and if β = 1 the worth is dYOL. Interventions that decrease YSL produce more worth if β is low, and interventions that increase YSL are favored if β is high. In the Birth cohort, HP/DP produced the most worth of all the single interventions if β <0.798, and ICU was most effective for higher values of β (calculations not shown). In the Retiree Cohort, the One-Shot intervention provided the most worth for β <0.741, and the ICU intervention was better for higher values of β. HP/DP was better than ICU for β < 0.606. Thus, the "best" intervention depends strongly on β, the relative worth to society of an additional YSL.

Although Column 6 allows us to compare the worth of interventions that were all improved by the same factor, α = .10, the One-Shot intervention is not a function of α, and so cannot fairly be compared to the others. To improve comparability, column 7 shows the value of α that would be needed for each intervention to produce the same number of worth-adjusted years as the One-Shot intervention. For example, in column 6 of Table 3 for the Retirees, the worth of One-Shot is .4180 and the worth of HP/DP is only .3590. To increase the worth of HP/DP to the One-Shot level, we must multiply its worth by .4180/.3590 = 1.164. Because incremental worth was found to be a linear function of α through the origin, the α that will achieve this change is 1.164 * .10 = .1164, which is tabled in Column 7. In the Birth cohort, One-Shot is equivalent to an α of about 0.01, while in the Retiree cohort it is equivalent to α between 0.1 and 0.2. Lower values in Column 7 are preferred, because they indicate that a smaller dose of the row intervention is needed to be equivalent to the One-Shot intervention. In both cohorts, HP/DP is the best single intervention that modifies probabilities and HP/DP + ICU is the best over-all intervention, in the sense of achieving the specified worth with the smallest amount of change in the transition probability (the smallest value of α).

Column 7 shows the required α when the initial conditions are the same as the U.S. population, and β = 0.5. More generally, if π is the proportion who are healthy at baseline,

αrequired=[π(dYHLH1+β∗dYSLH1)+(1−π)(dYHLS1+β∗dYSLS1)]∗.10π(dYHLHj+β∗dYHLHj)+(1−π)(dYHLSj+β∗dYSLSj),
 MathType@MTEF@5@5@+=feaafiart1ev1aaatCvAUfKttLearuWrP9MDH5MBPbIqV92AaeXatLxBI9gBaebbnrfifHhDYfgasaacH8akY=wiFfYdH8Gipec8Eeeu0xXdbba9frFj0=OqFfea0dXdd9vqai=hGuQ8kuc9pgc9s8qqaq=dirpe0xb9q8qiLsFr0=vr0=vr0dc8meaabaqaciaacaGaaeqabaqabeGadaaakeaaiiGacqWFXoqydaWgaaWcbaGaemOCaiNaemyzauMaemyCaeNaemyDauNaemyAaKMaemOCaiNaemyzauMaemizaqgabeaakiabg2da9maalaaabaGaei4waSLae8hWdaNaeiikaGIaemizaqMaemywaKLaemisaGKaemitaW0aaSbaaSqaaiabdIeaijabigdaXaqabaGccqGHRaWkcqWFYoGycqGHxiIkcqWGKbazcqWGzbqwcqWGtbWucqWGmbatdaWgaaWcbaGaemisaGKaeGymaedabeaakiabcMcaPiabgUcaRiabcIcaOiabigdaXiabgkHiTiab=b8aWjabcMcaPiabcIcaOiabdsgaKjabdMfazjabdIeaijabdYeamnaaBaaaleaacqWGtbWucqaIXaqmaeqaaOGaey4kaSIae8NSdiMaey4fIOIaemizaqMaemywaKLaem4uamLaemitaW0aaSbaaSqaaiabdofatjabigdaXaqabaGccqGGPaqkcqGGDbqxcqGHxiIkcqGGUaGlcqaIXaqmcqaIWaamaeaacqWFapaCcqGGOaakcqWGKbazcqWGzbqwcqWGibascqWGmbatdaWgaaWcbaGaemisaGKaemOAaOgabeaakiabgUcaRiab=j7aIjabgEHiQiabdsgaKjabdMfazjabdIeaijabdYeamnaaBaaaleaacqWGibascqWGQbGAaeqaaOGaeiykaKIaey4kaSIaeiikaGIaeGymaeJaeyOeI0Iae8hWdaNaeiykaKIaeiikaGIaemizaqMaemywaKLaemisaGKaemitaW0aaSbaaSqaaiabdofatjabdQgaQbqabaGccqGHRaWkcqWFYoGycqGHxiIkcqWGKbazcqWGzbqwcqWGtbWucqWGmbatdaWgaaWcbaGaem4uamLaemOAaOgabeaakiabcMcaPaaacqGGSaalaaa@A06D@

where dYHL_Hj _denotes the change in YHL caused by intervention j if everyone is healthy at baseline, dYHL_Sj _is the change if everyone is sick at baseline, and dYHL_H1 _and dYHL_S1 _refer to the One-Shot intervention. This equation was used to calculate the required α for different combinations of π and β. Table 4 indicates which of the simple interventions has the lowest required α for different values of π and β. For the birth cohort, HP/DP is best for β < 0.4, ICU is best for β > 0.7, and HP/DP is preferred to ICU for higher values of π for β between .4 and 0.7. For the Retiree cohort, the Treatment intervention is preferred when both β and π are low; that is, when the initial population is less then 40% healthy and the relative worth of an additional YSL is low. The preferred interventions for negative values of β were the same as those for β = 0, and are not shown separately. Table 4 thus shows that HP/DP is not always the best intervention, although under the standard configuration (π = .98 or .80, β = .5) HP/DP would be preferred. For different values of π or β, an ICU or a Treatment intervention might be preferable. The Safety intervention is never the best. Table 4 is correct for all values of α and λ, but does not permit assessment of the combined interventions.

**Table 4:** Which Simple Intervention* achieves the same Worth as One-Shot with the smallest α for different initial conditions and values of β

		**Proportion Healthy at Baseline, π**									
		0	0.1	0.2	0.3	0.4	0.5	0.6	0.7	0.8	0.9

**Birth cohort**											
β = 0		H	H	H	H	H	H	H	H	H	H
0.1		H	H	H	H	H	H	H	H	H	H
0.2		H	H	H	H	H	H	H	H	H	H
0.3		H	H	H	H	H	H	H	H	H	H
0.4		I	H	H	H	H	H	H	H	H	H
0.5		I	H	H	H	H	H	H	H	H	H
0.6		I	I	I	I	H	H	H	H	H	H
0.7		I	I	I	I	I	I	H	H	H	H
0.8		I	I	I	I	I	I	I	I	I	I
0.9		I	I	I	I	I	I	I	I	I	I
1.0		I	I	I	I	I	I	I	I	I	I
**Retiree cohort**											
β = 0		T	T	T	T	H	H	H	H	H	H
0.1		T	T	T	T	H	H	H	H	H	H
0.2		T	T	T	T	H	H	H	H	H	H
0.3		T	T	T	T	H	H	H	H	H	H
0.4		I	I	I	I	I	I	H	H	H	H
0.5		I	I	I	I	I	I	I	I	H	H
0.6		I	I	I	I	I	I	I	I	H	H
0.7		I	I	I	I	I	I	I	I	I	H
0.8		I	I	I	I	I	I	I	I	I	I
0.9		I	I	I	I	I	I	I	I	I	I
1.0		I	I	I	I	I	I	I	I	I	I

* H is HP/DP, T is Treatment, I is ICU, and S is Safety (never the best). The parameter π is the baseline proportion healthy, and β is the relative worth of a year of sick life. For example, if π = .8 and β = .5, in the Retiree cohort the HP/DP intervention had the lowest required α, as was also seen in column 7 of Table 3.

Another important consideration is the cost of implementing and maintaining the interventions. This requires specifying the cost of an intervention that improves a transition probability by 100*α% (or the cost of making 100*λ% of the sick persons healthy at baseline). In Appendix 3 we show that under some assumptions, one intervention is cost-effective relative to another intervention if the ratio of their costs is lower than the inverse of the ratio of their required α. As we have no information about the costs of these hypothetical interventions, further discussion of cost-effectiveness is limited to the examples in the discussion section.

## Discussion

This paper makes several contributions to the literature. We conceptualized the population as a system with three (or more) health states. Because the only way to alter a system is to change the initial conditions or the transition probabilities, all public health interventions must make one or more of these changes. This allows us to categorize types of interventions and compare them in a systematic manner. We incorporated β, the relative worth of an additional year of sick life, into the calculations and found it to be influential in determining the relative performance of various interventions. By standardizing the outputs of all interventions to the One-Shot intervention, we obtained a fair comparison of the different interventions. And finally, we found that the amount of improvement in YHL, YSL, and YOL is a linear function of α through the origin (for |α| <0.3), which allowed us to calculate multi-state life tables for only a few cases but to extend the results to many other situations.

Multi-state life table methods have been used elsewhere to estimate the consequences of modifying the transition probabilities, decreasing the prevalence of certain diseases or causes of death, or of meeting the healthy People 2000 objectives, on mortality and morbidity [14-23] and medical expenditures [24]. This literature usually involves only older adults (65 or older), and does not deal specifically with the type of interventions that might achieve such changes.

General features of the interventions are described next, followed by some specific examples that illustrate the use of this material.

### Features of the Interventions

Figures 2 and 3 show that in this country there may be little room for improvement of either the transition probabilities or the initial conditions under age 40. It will be more effective to target the health of middle-aged and older adults, or subsets of the younger population where the prevalence of sickness and the probability of becoming sick or dying are higher. All interventions improved YHL and YOL, but interventions that aimed to increase YOL also increased morbidity and medical expenditures. Table 4 shows which types of intervention are most effective, and also how strongly this conclusion depends on π and β.

The One-Shot intervention is equivalent to replacing the dashed lines in Figure 3 with the solid lines (making all the sick persons healthy at baseline). The area between the topmost solid and dashed curves in Figure 3 (multiplied by the proportion of Birth cohort who are initially sick) is the additional YHL associated with the One-Shot intervention. Clearly, the effect of One-Shot on YHL is short-term, and is small if there are few sick persons at baseline. One-Shot thus performs better in the Retiree cohort (see Figure 4). The effect of making only half of the sick persons healthy at baseline (λ = .5) can be obtained by halving the values for One-Shot in Table 3.

The HP/DP, Treatment, and One-Shot interventions work by keeping or making more persons healthy, thus directly improving YHL and YSL. They indirectly improve YOL because healthy persons have a lower mortality rate. The ICU intervention keeps sick persons from dying, thus directly increasing YOL and YSL, and indirectly increasing YHL because persons saved from death may later become healthy. The Safety intervention directly increases YOL and YHL, but also somewhat surprisingly increases YSL, because the persons saved by the intervention then live long enough to accumulate YSL instead of dying young. The net effect of these considerations is that the former three interventions decrease medical expenditures, while the latter two increase them.

The two combined interventions performed differently. The worth of HP/DP + One-Shot (0.7925 in the Retiree cohort) was slightly better than the sum of the worth of HP/DP and One-Shot separately (0.3590+0.4180 = 0.7770). This apparent synergy may occur because the two interventions act on different parts of the distribution, with One-Shot initially increasing the number of healthy persons for the HP/DP intervention to keep healthy. The HP/DP + ICU intervention provided slightly less worth than the sum of its components, possibly because the HP/DP intervention kept people healthy, leaving the ICU intervention with fewer sick persons to save from death. HP/DP+ICU increased medical expenditures even though it decreased YSL, apparently because it made relatively large changes in YOL but relatively small decreases in YSL. It is easy to evaluate the addition of One-Shot to the TX, Safety, and ICU interventions, as explained in Appendix 2. We presented only HP/DP + ICU in Tables 2 and 3 because it was better than the other combinations (for β = .5). Combining different interventions would require additional life table calculations.

It is possible to improve the estimates of the transition probabilities and initial conditions, at least in the U.S., where many public population-based longitudinal datasets that ascertain death are available for subpopulations of interest. We are not aware of any source for β, the relative worth to society of an additional year of sick life. This information is needed because the choice of the best intervention is sensitive to β. There is evidence that β < 1 because we invest in treatments for health problems that are not life-threatening. Recent public discussions about assisted suicide and withdrawal of life support from persons in a persistent vegetative state suggest that β > 0. As suggested in Table 4, the usual public health emphasis on prevention programs makes an implicit assumption that β < .798 in the Birth cohort and β < .606 in the Retiree cohort; otherwise, public health would emphasize keeping sick persons from dying (ICU), which provides the most worth-adjusted years of life when β is large. We showed results for β = .5, which was an arbitrary choice. Interestingly, the method used by the National Center for Health Statistics to calculate years of healthy life (by a different method from that reported here) are consistent with a β of about .5 for older adults [25]. Further research is needed in this area.

As discussed in Appendix 3, costs of the interventions are crucial for decision making, but they are not known or obvious for the hypothetical interventions we have considered. When these costs are known, the least expensive intervention in column 7 of Table 3 would be the most cost-effective.

### Examples

Here we present examples that use the information in Tables 2 and 3 under the standard parameter configuration (λ = 1, α = .1, β = .5, π = .98 and .80), and also for some different parameter values. (Appendix 2 explains the calculations for other parameter values).

Suppose that a public health department could afford a new program either to (A) make half of the sick 65-year-olds healthy immediately (One-Shot with λ = .5), or (B) decrease the probability of becoming sick each year by 5% in the Birth cohort (HP/DP with α = .05), and also assume that β = .5. From Table 3, the worth of (A) is .4180* λ = .2090 worth-adjusted years, and the worth of (B) is α/.10*.7480 = .3740. Other things equal, option B would be preferred. However, if instead π = .90 for Birth cohort and π = .50 for the Retiree cohort, the worth of A and B would be .5224 and .3728 respectively, and option A would be chosen. Or, if β was 0.7 instead of 0.5, the worth would be .1856 and .3042, and option B would be chosen. The choice of intervention is sensitive to the parameter values.

We next consider an existing intervention, "Welcome to Medicare" (WTM), which covers a one-time preventive physical exam within the first six months of enrolment in Part B. The exam includes a thorough review of the enrollee's health, education and counseling about preventive services such as screenings and shots, and referrals for other care. This is similar to our HP/DP+One-Shot intervention, where the population consists of all 65-year-olds who elect Part B coverage. Here, λ is the proportion of the sick persons who are returned to the healthy state at baseline, and α is the subsequent decrease in the probability of getting sick, due to the prevention. Table 3 shows that if λ = 1 and α = .10 (if all of the initially sick enrollees become healthy and the probability of becoming sick decreases by 10%), this would increase YHL by 1.041 years and increase YOL by .544 years, while decreasing YSL by .497 years. There would be essentially no change in future medical expenditures, even though enrollees would live longer.

If the average incremental cost of the WTM program was $1000 per person, the cost per year of life saved would be $1000/.544 or about $2000 per additional year of life. If WTM was less effective, with λ = .08 and α = .008, YOL would increase by .021 years, and an additional year of life would cost about $50,000, which is a common threshold for cost-effectiveness. Under these assumptions, Welcome to Medicare is cost-effective for the configuration λ = .08 and α = .008, and for larger values of either parameter. It would be interesting to estimate these parameters for persons with and without Part B coverage, to provide further information about cost-effectiveness.

Modifying π permits assessment of natural interventions such as a pandemic that sickened all newborns. Figure 3 (for all sick at birth, or π = 0) shows that the Birth cohort would return to its equilibrium number of healthy and sick by about age 5. The area between the top solid and dashed lines is the loss in YHL due to the pandemic. Using the standard configuration for the other parameters, the Status Quo would be 64.52 YHL (vs. 67.80 in Table 2 line 1), and 74.46 YOL (vs. 77.3). The effect of such a pandemic would thus be the loss of 3.28 years of healthy life and 2.84 years of life. One-Shot is the best single intervention, resulting in 3.14 additional worth-adjusted years if λ = 1.0. A pandemic that sickened only half of the newborns (π = .5) would decrease YHL by 1.61 years and YOL by 1.38 years.

The tabled results can also be used to estimate the effect of "negative" interventions. For example, if the intervention was a chronic illness that lowered the probability of recovering from illness by 10% at all ages, that would be the same as the Treatment intervention with α = -0.10, and the effects of the various interventions would be the values in Table 3 (columns 2–4 and 6) multiplied by -1. For the Birth cohort, this illness would decrease YHL by .858 years, increase YSL by .554 years, and decrease YOL by .303 years.

The tabled results, and a spread sheet available from the authors that allows for different parameter values, should be useful in assessing other hypothetical or existing programs, or to consider the effect of modifying some of the parameters. More complex models may be needed to compare specific interventions.

### Limitations

The interventions considered are of course unrealistic. There are probably few interventions that affect only a single transition probability, or that could achieve the same improvement at all ages. A package of interventions would likely be needed, perhaps varying with age. We assumed that α was the same for each age, but the life table calculations for age-varying values of α would be straightforward. Only small values of α were considered, but this may be appropriate. The examples are simplistic, but suggest some applications of this work.

For simplicity, we considered only three health states, but more complex models are possible. The states were defined based on self-rated health, which has face validity as an important descriptor of the health of a population, and is a well known correlate of mortality and of most other important health variables [26]. (The strong association of self-rated health with mortality can be seen in Figures 1 and 2). The states could instead have been based on other measures such as activities of daily living. If so, we could have estimated active life expectancy rather than years of healthy life [27]. The resulting three-state system would have different transition probabilities and a different initial distribution. The conclusions about the relative performance of the different intervention types would probably have been similar, but this must be verified elsewhere.

We ignored gender and race, in the interest of simplicity. Gender- or race-specific transition probabilities would have resulted in different YHL, YSL, and YOL [28] but not, we believe, in different findings about the relative behavior of the interventions. As with all life table calculations, the transition probabilities at later ages may not be appropriate for persons born today, and these estimates cannot be exact.

Medical expenditures were estimated from a single year of MEPS data, and were extrapolated for ages 85 to 100. Small changes in expenditures in Table 3 are well within the range of error. Some of the interventions affected lifetime medical expenditures. However, many of these savings (or additional expenditures) would disappear under the traditional 3% discounting for costs accrued over time. We have not examined whether d$ is linear in α through the origin. If it is, then changes in medical expenditures can also be predicted for many configurations.

In this paper, we must assume that the interventions do not change the heterogeneity within the healthy and sick states at each age, relative to the general population. The small values of α that we used should minimize this problem. For larger values of α, heterogeneity may be a problem, requiring more complex models. Appendix 4 provides additional discussion of heterogeneity.

## Conclusion

Although a good deal of research has considered the mortality and disability patterns of cohorts, our paper may be the first to present transition probabilities across the entire age range, and to examine the effects of specific types of interventions on years of healthy life. Some insights have been gained as to how different interventions work to improve the health of the public. Health promotion and disease prevention strategies had favorable performance under most conditions, but ICU and Treatment were sometimes better. The Safety intervention would be implemented by passing and enforcing laws, rather than by intervening directly on healthy and sick persons. It might be cost-effective relative to the other interventions even though it was never selected in Table 4. The importance of β suggests that it is time for a national discussion of the value to society of an intervention that increases survival by increasing morbidity.

The small number of sick persons at any age is a tribute to today's public health strategies. These results suggest how the situation might be improved further by appropriate public health intervention strategies, especially at older ages.

## Competing interests

The author(s) declare that they have no competing interests.

## Authors' contributions

PD designed the paper, performed the analyses, and is the primary author. AD provided data and commented on early drafts. AN commented on early drafts. LC provided data and commented on early drafts. All authors read and approved the final manuscript.

## Pre-publication history

The pre-publication history for this paper can be accessed here:



## Supplementary Material

Additional file 1 Transition Data and Multi-state Life tables. Appendix 1. Additional information about the source of data used in calculating transition probabilities, and an example of a multi-state life tagble calculation Click here for file

Additional file 2 Appendix 2. Using different parameter values. Additional information about how to use the data in the text to perform calculations using different parameter values. Click here for file

Additional file 3 Appendix 3. Cost and cost-effectiveness. Additional discussion of the effect of the cost of implementing and maintaining the intervention on the findings, and issues involving a potential cost-effectiveness analysis. Click here for file

Additional file 4 Appendix 4. Heterogeneity. Additional discussion of how heterogeneity within the various health states might affect the findings. Click here for file
