# Supplementary material for: The effect of different public health interventions on longevity, morbidity, and years of healthy life
Source: BMC Public Health. 2007 Apr 5;7:52. doi: 10.1186/1471-2458-7-52 (PMC1853080; doi:10.1186/1471-2458-7-52)
Supplement: Additional file 1 — Transition Data and Multi-state Life tables. Appendix 1. Additional information about the source of data used in calculating transition probabilities, and an example of a multi-state life tagble calculation [file 1471-2458-7-52-S1.doc]

**Appendix 1: Transition data and multi-state life tables**

Age-specific transition probabilities were developed for self-rated health (is your health excellent, very good, good, fair or poor?), based on three large datasets. The Cardiovascular Health Study (CHS) is a population-based longitudinal study of 5,888 adults aged 65 and older at baseline, described in detail elsewhere [29,30]. Self-rated health was obtained every 6 months (to date), beginning in 1990. The 5,888 subjects contributed about 150,000 transition pairs (two self-rated health values for the same person measured one year apart). Similar transition information was also taken from two large national surveys, the Medicare Current Beneficiary Study (MCBS) [31] (98,000 transition pairs) and the Medical Expenditures Panel Survey (MEPS) [32] (224,000 transition pairs).

Readers unfamiliar with multi-state life table calculations, may be interested in the following example. Consider a cohort with 80,000 healthy and 20,000 sick persons at age 65 (similar the U.S. distribution). Figure 1 shows the estimated probabilities of moving from one state at age 65 to another state at age 66. The probabilities indicate that .09*80,000=7200 healthy persons are expected to be sick one year later, 800 will be dead, and 72,000 will still be healthy. Of the 20,000 sick persons, 12,400 will remain sick, 6600 will become healthy, and 1000 will die. Thus, at age 66, there would be 72,000+6600=78,600 healthy persons, 19,600 sick persons, and 1800 dead persons. The process can be repeated, using age-specific probabilities, until all subjects have died. The person-years spent in the healthy or sick states can then be summed, to yield estimates of the future years of healthy life (YHL), years of sick life (YSL), and years of life (YOL).
