# Supplementary material for: The effect of different public health interventions on longevity, morbidity, and years of healthy life
Source: BMC Public Health. 2007 Apr 5;7:52. doi: 10.1186/1471-2458-7-52 (PMC1853080; doi:10.1186/1471-2458-7-52)
Supplement: Additional file 2 — Appendix 2. Using different parameter values. Additional information about how to use the data in the text to perform calculations using different parameter values. [file 1471-2458-7-52-S2.doc]

**Appendix 2: Using different parameter values**

In Table 2, the data in columns 2-7 were calculated from a multi-state life table program for Status Quo, HP/DP, Treatment, Safety, ICU, and HP/DP+ICU. The remainder of the lines and columns can then be calculated from these lines using simple spreadsheet calculations. The One-Shot columns 2-4 are the same as Status Quo 2-4. One-Shot columns 5-7 are a mix of the healthy at baseline and sick at baseline columns for Status Quo, depending on the value of λ. For example, column 5 for One-Shot = λ*(column 2 for Status Quo) + (1- λ)*column 5 for status quo. Similarly, HP/DP+One-Shot has the same entries as HP/DP columns 2-4, and is a mix in columns 5-7. One-Shot can easily be added to the Treatment, ICU, and Safety in the same way. This completes columns 2-7 for Table 2.

Columns 8-10 are calculated as weighted sums of columns 2-7; for example, column 8 is π*(column 2) + (1- π)* (column 5). In Table 3, columns 2-4 are calculated by subtracting the Status Quo (line 1) from the other values in Table 2 columns 8-10. Column 6 in Table 3 is calculated as (column 2) + β * (column 3). Column 7 is calculated from column 6, and the intervention with the lowest value in column 7 is a single entry in Table 4, for the appropriate value of π and β (HP/DP is best in both cases). Programming these calculations into a spreadsheet will replicate the information in Table 2 and 3. Varying the values of π and β in the spreadsheet will provide information for the additional cells in Table 4.

We next examine the effect of varying λ and α. The effect of making only, say, half of the sick persons healthy at baseline in the One-Shot intervention (λ = .5) would be to multiply λ*the values for One-Shot in Columns 2, 3, 4, and 5 of Table 3. The effect of improving the transition probabilities by a value of α different from .10 is to multiply columns 2, 3, and 4 (not 5) in Table 3 by α/.10; this is appropriate for the rows labeled HP/DP, Treatment, ICU, Safety, and HP/DP+One-Shot. (Column 5, d$, is presented only for the standard configuration. It is a constant multiple of λ, but we have not studied whether it is also a constant multiple of α).

Given this ability to create tables for a great variety of situations, many comparisons can be made. A spreadsheet that calculates Table 2 and Table 3 for different values of the parameters is available from the authors.
