# Supplementary material for: The effect of different public health interventions on longevity, morbidity, and years of healthy life
Source: BMC Public Health. 2007 Apr 5;7:52. doi: 10.1186/1471-2458-7-52 (PMC1853080; doi:10.1186/1471-2458-7-52)
Supplement: Additional file 3 — Appendix 3. Cost and cost-effectiveness. Additional discussion of the effect of the cost of implementing and maintaining the intervention on the findings, and issues involving a potential cost-effectiveness analysis. [file 1471-2458-7-52-S3.doc]

**Appendix 3: Cost and cost effectiveness**

The main text does not discuss the cost of implementing and maintaining the interventions. Doing so requires specifying the cost of an intervention that improves a transition probability by 100*α% (or the cost of making 100*λ% of the sick persons healthy at baseline). Here, we assume that the cost of the intervention is proportional to the amount of change required in the transition probabilities, α. For instance, the cost to achieve an improvement of α_h_ using HP/DP is assumed to be C_h_* α_h_, and the cost of achieving an improvement of α_i_ using ICU is C_i_* α_i_. From column 7 of Table 3, the cost to achieve .0625 worth-adjusted years is C_h_ * .0084 using HP/DP and is C_i_ * .0135 using ICU. These two costs are the same if C_h_ = C_i_ * .0135/.0084 = C_i_ * 1.607, and HP/DP is cost effective relative to ICU if C_h_ / C_i_ < 1.607. Similarly, in the Retiree cohort, if the cost of HP/DP is less than .1357/.1164 = 1.166 times the cost of ICU, or less, then HP/DP is more cost-effective. Interventions are cost-effective relative to One-Shot if the ratio of costs is less than the value in column 7. For example, in the Retiree cohort, One-Shot is cost effective compared to HP/DP if One-Shot costs less than 0.1164 times the cost of HP/DP. Although the costs of a particular implementation are unknown in this exercise, these relative numbers may be useful. It is clear, for instance, that in the Birth cohort, One-Shot would not be chosen unless it cost less than 1% as much as another intervention.

The costs associated with an intervention are not obvious. HP/DP may have low costs per person, but as it is applied to all healthy persons it may be expensive in total. Treatment and ICU may have high per-person costs, but they are applied only to the smaller number who are sick. It may be expensive to make everyone healthy at baseline, particularly if discounted costs are calculated, because the One-Shot intervention incurs costs up front, while other interventions will accrue costs (and benefits) over time. On the other hand, One-Shot accrues most of its benefit early on, and discounting the benefit (which is usually done when costs are discounted) may favor One-Shot for that reason. In Table 4, the Safety intervention was never selected; that is, for all values of β or π there was another intervention that could achieve as much worth with a smaller α. However, as the Safety intervention might be implemented by passing and enforcing laws, rather than by intervening directly on healthy and sick persons, it remains possible that Safety would be cost-effective relative to the other interventions. Additional research is needed into the costs of such interventions, to explore whether the savings in future medical expenditures seen in Table 3 might offset some of the costs of the interventions.
