# Supplementary material for: The effect of different public health interventions on longevity, morbidity, and years of healthy life
Source: BMC Public Health. 2007 Apr 5;7:52. doi: 10.1186/1471-2458-7-52 (PMC1853080; doi:10.1186/1471-2458-7-52)
Supplement: Additional file 4 — Appendix 4. Heterogeneity. Additional discussion of how heterogeneity within the various health states might affect the findings. [file 1471-2458-7-52-S4.doc]

**Appendix 4: Heterogeneity**

An important consideration in transition models is heterogeneity, the amount by which persons in the same state differ in their transition probabilities. The healthy state, for example, includes people who rated their health as excellent, very good, or good, and each of these states has different transition probabilities [28]. Some currently healthy persons have been healthy for years, while others might have been sick in the previous year; the latter group has less favorable transition probabilities than the former (data not shown). Some persons have acute or chronic diseases, and others do not. It is clear that the healthy and sick states are heterogeneous.

This heterogeneity does not affect the estimates made under the Status Quo, as these estimates are calculated from the average transition probabilities in each state, sometimes referred to as the “cohort” transition probabilities [33]. The average probabilities were estimated separately for each age from population-based data and should be valid for the mix of persons at this age in the general population. Heterogeneity is, however, a potential problem for the interventions. For example, if the ICU intervention kept the sickest of the sick alive, the average probability of recovery to the healthy state would be lower than what was estimated in the general populations.

We briefly considered a 5-state model where the sick state was divided into two sub-states, “newly sick” and “still sick”, and similarly the healthy state was divided into newly healthy and still healthy. We estimated the transition probabilities among these 5 states, and performed the life table calculations for the Retiree cohort using large values of α. Under the Status Quo, about 63% of the YSL were spent in the still sick category. The One-Shot intervention (moving all the sick at baseline into newly healthy) decreased that percentage to 60%, the ICU (with α=.43) increased it to 68%, and the Safety intervention (with α=.33) decreased it to 62%. These interventions, with large values of α and λ, thus did change the mix slightly.

In this paper, we must assume that the interventions act in such a way as not to change the heterogeneity within the healthy and sick states at each age, relative to the general population. This could be true if, for example, the One-Shot intervention moved people into the newly healthy and still healthy categories in proportion to the population ratios, or if the intervention improved the individual transition probability for every person in the state by the same factor, α. It is also likely that heterogeneity will not have much effect if α is small, which is the situation that we have reported. For large values of α, heterogeneity may be a problem, requiring more complex models.
